# Mobile Affinity Selection Chromatography Analysis of Therapeutic Monoclonal Antibodies

**DOI:** 10.1021/acs.analchem.3c02180

**Published:** 2023-10-26

**Authors:** Meena L. Narsimhan, Jinhee Kim, Nathan A. Morris, Mary A. Bower, Harsha P. Gunawardena, Eric Bowen, Fred E. Regnier

**Affiliations:** † Novilytic, LLC, 1281 Win Hentschel Boulevard, West Lafayette, Indiana 47906, United States; ‡ Janssen Research & Development, The Janssen Pharmaceutical Companies of Johnson & Johnson, Spring House, Pennsylvania 19477, United States

## Abstract

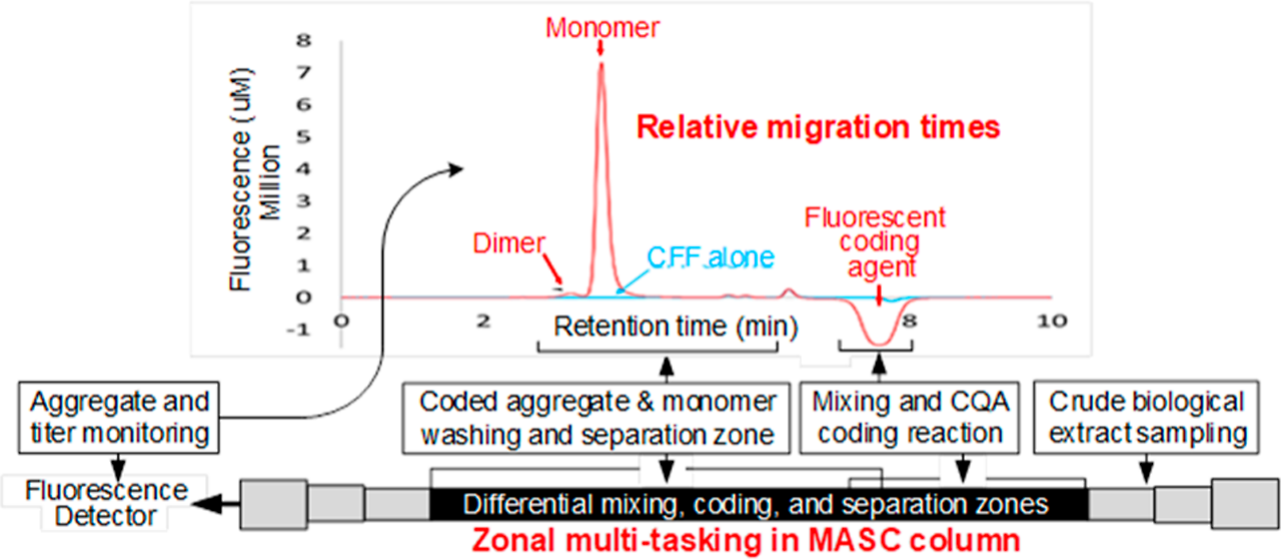

Federal regulatory agencies require continuous verification of recombinant therapeutic monoclonal antibody (mAb) quality that is commonly achieved in a two-step process. First, the host-cell proteome and metabolome are removed from the production medium by protein A affinity chromatography. Second, following recovery from the affinity column with an acidic wash, mAb quality is assessed in multiple ways by liquid chromatography–mass spectrometry (LC–MS). However, lengthy sample preparation and the lack of higher-order structure analyses are limitations of this approach. To address these issues, this report presents an integrated approach for the analysis of two critical quality attributes of mAbs, namely titer and relative aggregate content. Integration of sample preparation and molecular-recognition-based analyses were achieved in a single step utilizing an isocratically eluted mobile affinity selection chromatography (MASC) column. MASC circumvents the protein A step, simplifying sample preparation. Within 10 min, (i) mAbs are fluorescently coded for specific detection, (ii) monomers and aggregates are resolved, (iii) the mAb titer is quantified, (iv) relative aggregate content is determined, (v) analytes are detected, and (vi) the column is ready for the next sample. It is suggested herein that this mode of rapid quality assessment will be of value at all stages of discovery (screening, clone selection, characterization), process R&D, and manufacturing. Rapid monitoring of variant formation is a critical element of quality evaluation.

## Introduction

Therapeutic monoclonal antibodies (mAbs) make up a family of recombinant immunoglobulin (IgG) proteoforms. A single host-cell gene, native or recombinant, can give rise to multiple structurally related forms of the mAb.^1^ This leads to a mixture of many proteoforms resulting from small changes in the production environment. These changes may in turn result in alterations in critical quality attributes (CQAs) within product proteoforms.^2,3^ The CQA used here defines features of a proteoform critical to its biological function. A CQA may positively or negatively impact the therapeutic efficacy and safety of a recombinant protein. Negative CQAs are those that compromise the product quality.

Recognizing the significance of this problem, the FDA introduced directives for continuous process verification in the production of therapeutic proteins in 1987.^4,5^ Their guidelines involved identifying and monitoring CQAs that define product safety and efficacy at all stages of development and production, the objective being to recognize patterns of process deviation within a time frame that allows remediation and portends future process deviations. The focus here is on the identification and assay of CQAs that define the structure and quality of recombinant therapeutic proteins, specifically mAbs, as suggested by the FDA for quality evaluation.^6^

The most widely used analytical route to mAb safety and efficacy appraisal has focused on the identification and the quantification of posttranslational modifications (PTMs)^7^ such as deamidation,^8^ amino acid side chain oxidation,^9,10^ disulfide scrambling,^11^ lysine glycation,^12^ and glycosylation^13^ that have all been implicated in mAb quality. PTMs are typically monitored by bottom-up and middle-down liquid chromatography–mass spectrometry (LC–MS). The primary structure of proteins is thus obtained by correlating the mass of gas phase fragment ions of polypeptides with DNA sequences in genomic libraries.^14^ Although mass determinations are achieved in milliseconds, multiple time-consuming steps of preliminary sample purification, proteolysis, and resolution of polypeptide fragments precede structure elucidation and delay decision making. Newer methods of MS analysis of intact mAbs are fast and have shown high throughput.^15^ However, they may still be more difficult to implement in pharma process development or in continuous process verification due to the higher cost and expertise required.

A problem with the PTM analysis approach is that only a fraction of the mAb variant pool impacts mAb quality, with a varying probability of occurrence.^16−18^ The yield of useful data relative to the effort invested is low, and a more direct approach is needed. We posit here that monitoring mAb aggregation is an attractive alternative; wherein the term aggregate refers to a dimer, trimer, tetramer, or other species that can be separated by size-exclusion chromatography (SEC). PTM variants such as those listed above arise during mAb production, purification, and formulation and often lead to the production of immunogenic or toxic aggregates.^19^ Due to the importance of aggregate and titer as CQAs, aggregate and titer monitoring by LC provides an early, inexpensive, and convenient method for estimation of quality loss, precluding the need for routine mass spectral analyses. Being a serial assay method, it allows rapid data-dependent decision making (i) in either a process development or production environment, (ii) with a single analytical platform, and (iii) requires limited sample preparation. For high throughput, the LC method should also specifically detect the mAb among the 1000 to 1500 host-cell protein background of cell-free fermentation media.

To meet these requirements, we describe here a rapid mAb titer and aggregate analyses through an adaptation of mobile affinity selection chromatography (MASC).^20,21^ MASC is a three-phase chromatographic process that differs from conventional two-phase chromatography in having a soluble third phase consisting of an affinity selector. The analyte of interest partitions with the mobile affinity selection phase (P_as_^*^) and the stationary phase through different mechanisms.^22,23^ In effect, two modes of chromatography, molecular sieving and affinity selection, are achieved simultaneously in MASC. Substances of no interest partition solely with the stationary phase, while those of great interest partition with both the mobile affinity selection phase and the stationary phase. We describe here a MASC method that is fast and eminently suited for rapid quality monitoring.

Titer and aggregate ratio analyses of mAbs were used to develop and validate the method. In this approach, the third phase is a fluorescently labeled affinity selector of low molecular weight that is included in the mobile phase. The third phase is designed to bind with high selectivity and affinity to a CQA in the analyte proteoform family, forming fluorescent complexes that are transported through the column and resolved by a molecular sieving mechanism. Fluorescently labeled proteoform complexes eluted from the MASC column are detected with a flow-through postcolumn detector. Based on the use of fluorescence detection, the new methods described here will be referred to as “*MASC luminon assays.*”^24^

## Experimental Section

### Reagents and Supplies

Monoclonal antibodies (mAbs) used in MASC luminon assay validation were (i) NIST (National Institute of Standards and Technology) Monoclonal Antibody Reference Material 8671 (NmAb), (ii) rituximab biosimilar, (iii) denosumab biosimilar, and (iv) nivolumab biosimilar. The biosimilars were purchased from Ichorbio (UK). Mobile phase buffer salts and the fluorescent mobile affinity selector reagent for the MASC luminon assay were from the Proteometer-L kit (Novilytic) and were employed to prepare mobile phase L-MP according to the manufacturer’s instructions. l-His buffer was 12.5 mM l-histidine buffer, pH 6.0. PBS was phosphate buffered saline, pH 7.2. Analytical reagent grade chemicals were used throughout. Cell-free filtrate (CFF) was prepared from spent growth medium of cultured ExpiCHO-S Cells (ThermoFisher Scientific) that were grown for 8 days in shake flasks in ExpiCHO Expression Medium (ThermoFisher Scientific) according to the guidelines provided by the manufacturer. Cell viability was 93% and cell density was 8.09 × 10^6^/mL at harvest. Cells and particulate debris were removed by 4000*g* centrifugation at 4 °C. The resulting growth medium was further clarified by passage through a 0.22-μm filter to yield CFF. CFF aliquots were stored at −80 °C until use. CFFs containing the therapeutic bispecific antibody (bsAb) were obtained from bioreactor runs at Janssen. Protein Lo-bind tubes (Eppendorf) were used for sample storage and dilution. High recovery autosampler vials were used throughout.

### Chromatographic Systems, Columns, and Software

MASC columns (7.8 mm × 150 mm, 2.7 μm, 300 Å) were components of a Proteometer-L Kit (Novilytic). The stationary phase consisted of silica particles with a neutral hydrophilic coating. All bsAb samples were analyzed on an Agilent 1290 Infinity II UHPLC system equipped with a 1290 Infinity II multisampler, a 1260 Infinity II quaternary pump, and a 1260 Infinity II fluorescence detector. Acquisition of the bsAb data was performed using MassHunter software, while peak processing and integration were performed with Qualitative Analysis Navigator software (ver. B08). All other analyses were performed on a Shimadzu Nexera X-2 UPLC/HPLC instrument equipped with a SIL-30AC autosampler or a Shimadzu LC-40 liquid chromatography system equipped with a SIL-40 autosampler. Both LC systems were fitted with a Shimadzu RF-20Axs fluorescence detector. When an absorbance detector was required, either a Shimadzu SPD-20A UV or a SPD-40 PDA was used. The dead volume on both systems was less than 40 μL. Data acquisition and instrument control were performed using LabSolutions software. Peak integration was performed by using the i-PeakFinder algorithm.

### Sample Preparation

Unless stated otherwise, mAbs were diluted to 1 mg/mL in PBS, l-His buffer, or CFF for analyses. Samples used in the MASC assay protocol were free of cells and particulate debris exceeding 220 nm in size. Injection volume limits were determined by the injection valve supplied with the liquid chromatography system, typically 0.1–100 μL with the instruments used. The sample volume limit was set at 5% of the column volume.

### bsAb Titer Using Protein A

CFFs of bioreactor samples (three wells of 300 μL per sample) were arrayed in 96-well MTP plates (ThermoFisher Scientific) and subjected to automated protein purification on a Microlab STAR liquid handling system (Hamilton) using PhyNexus columns (Biotage; 300 μL) with MabSelect SuRe LX resin (Cytiva; 20 μL). Equilibration, bind, wash 1, wash 2, wash 3, and elution steps were performed using the manufacturer suggested buffers, with back-and-forth cycles (1, 3, 2, 2, 3, and 3 cycles, respectively) with 0.2 mL/min flow rates and 20 s pauses after aspiration and elution. Each column was loaded with CFF (250 μL) from one MTP well, and bound IgG was recovered in a single elution using 300 μL of elution buffer. The eluate was neutralized to approximately pH 6 by addition of 15 μL of 1 M Tris buffer, pH 9.0, creating a total volume of 315 μL. Protein concentration was determined by measuring absorbance at 280 nm, from which the bsAb titer in CFF was extrapolated.

### Assay Design

Among the multiple objectives of this MASC luminon assay, the first was to circumvent the need for the removal of sample contaminants prior to analysis. This began with fluorescent labeling of the CQA of interest in analyte proteoforms (A_p_). The rationale is that contaminants such as host-cell proteins and metabolites will be invisible in fluorescence detection of analyte. Fluorescent encryption of A_p_ was accomplished with a synthetic, low molecular weight affinity selector (P_as_^*^) that binds noncovalently with high affinity to the CQA of interest in A_p_.

A second objective was to achieve all the aspects of the assay within a single MASC column. That includes mixing and coding of A_p_ contained in the sample with the P_as_^*^, size-based resolution of A_p_:P_as_^*^ complexes from nonanalytes, and transport to a flow-through fluorescence detector. The third objective was to resolve and quantify the P_as_^*^ labeled mAb monomers along with determination of the relative aggregate content in the mAb sample.

The latter two objectives were achieved by MASC through the use of a molecular sieving stationary phase in the column and a low molecular weight affinity selector in the mobile phase. Typically, therapeutic mAbs have an intact molecular mass of about 150 kDa while the affinity selector chosen was 1 to 2 kDa. The small affinity selector travels slower than the mAb proteoforms in a molecular sieving column. mAbs added to the system were rapidly mixed with P_as_^*^ affinity selector in the mobile phase, initiating A_p_:P_as_^*^ complex formation. Continual migration of A_p_ and A_p_:P_as_^*^ complexes into a constant concentration of P_as_^*^ in the mobile phase during transport through the MASC column enables complex formation to continue while reducing dissociation of the formed complexes. The small size of the affinity selector diminished peak broadening, thus enabling size-based resolution and quantification of the A_p_:P_as_^*^ complexes.

The fourth objective was to examine the degree to which the MASC luminon assay could address unique structural features encountered in therapeutic mAbs. Therapeutic mAbs belong to three structurally distinct subclasses, IgG1, IgG2, and IgG4, that share about 90% amino acid sequence homology and overall structure^25^ but have characteristic differences that are confined largely to (a) the hinge region separating the Fab arms from the C-terminal Fc domain and (b) the N-terminal region of the CH2 domain of Fc. These differences in structural features convey unique conformation, nonantigen binding functions, and aggregation propensity to each subclass.^26^ Therapeutic mAbs contain entirely human or both murine and human IgG sequences. Biosimilars of rituximab, denosumab, and nivolumab were employed to examine the impact of these structural attributes on the MASC luminon assay. Rituximab is an IgG1κ subclass antibody, being chimeric with a murine Fab region fused to the human Fc region.^27^ Denosumab is of the IgG2κ subclass and fully human.^28^ Nivolumab is in the IgG4κ subclass, being fully human and carrying an S228P mutation in the Fc region for added stability and reduced variability.^29^ Bispecific antibodies (bsAbs), which are IgGs engineered to bind two unique antigens but have the same structure as traditional therapeutic mAbs, were also examined by the MASC luminon assay since they are a focus of newer development initiatives of biopharmaceutical companies.

The fifth objective was to validate the assay in the crude samples. This was achieved using bioreactor samples during production of a bsAb and in mimics of fermentor-derived samples that were prepared by the addition of purified mAbs to CFF.

## Results

### Method Validation

The MASC luminon assay described here is directed toward analyses of therapeutic mAbs, specifically the mAb titer and relative aggregate content. Simultaneous quantification of the mAb titer and relative aggregate content was achieved by coding a structural attribute common to all proteoforms of human IgG with the fluorescently labeled affinity selector reagent (Proteometer-L Reagent). A general representation of the coding reaction is
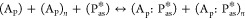
where A_p_ denotes all monomeric proteoforms of the analyte protein, (A_p_)_*n*_ signifies aggregated forms of the analyte protein, P_as_^*^ is the affinity selector, and fluorescence-coded forms of monomers and aggregates are represented by (A_p_:P_as_^*^) and (A_p_:P_as_^*^)_*n*_, respectively.

**Figure 1:**
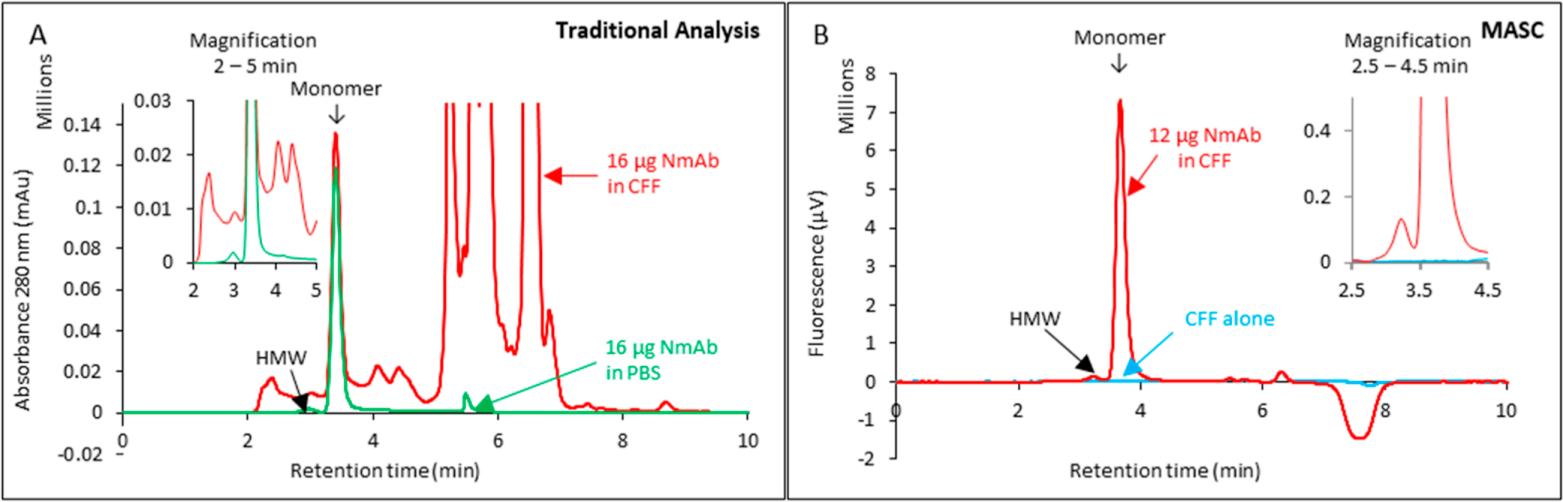
Value of the MASC method for mAb aggregate and titer analysis. (A) Traditional aggregate and titer analysis by SEC. Mobile phase, PBS. Flow rate: 1 mL/min. Detection method, absorbance at 280 nm. NmAb samples were diluted to 1 mg/mL in PBS or CFF for analysis. The overlaid chromatograms and the magnification (inset) illustrate interference by host-cell proteins that hamper quantification of HMW content and titer. (B) Aggregate and titer analysis by MASC. Mobile phase, L-MP from Proteometer-L kit. Flow rate, 1 mL/min. Detection method, fluorescence Ex. 450 nm Em.520 nm. NmAb sample was diluted to 1 mg/mL in CFF for analysis. An equal volume of CFF alone was injected for comparison. The overlaid chromatograms and the magnification (inset) illustrate no interference by host-cell proteins to hamper quantification. The portion from 5 to 8.5 min reveals some nonspecific signal related to the presence of CFF in the sample that does not interfere with the response of the analyte of interest. Difference in *y*-axis scales between Panels A and B illustrates the high sensitivity achieved by the MASC method.

Initial method validation experiments described herein were achieved with a NIST monoclonal antibody (NmAb) reference standard. The NmAb standard is a mixture of both monomeric and aggregate proteoforms that have been resolved and individually quantified by size exclusion chromatography (SEC) assays.^30,31^ Analysis was performed on NmAb samples diluted in buffer and on simulated fermentor samples created through the addition of known quantities of NmAb to the cell-free filtrate (CFF) of cultured, untransformed CHO cells. The rationale was that through fabrication with pure NmAb, the concentrations and quality of the samples analyzed in both matrices would be identical. Detection was first achieved by absorbance at 280 nm without fluorescence coding (Figure 1A). Aggregate and monomer proteoforms eluted from the SEC column in 3–4 min. Overlaid chromatograms of CFF-free and CFF bearing NmAb samples illustrate coelution of host-cell proteins with the NmAb standard, hampering quantification of the mAb titer and aggregate content. Resolution of the NIST mAb monomer and aggregates on the Novilytic MASC column and commercial 300 Å pore diameter SEC columns were similar. From this it is concluded that the mAb separation mechanism on the MASC column is by size exclusion.

The CFF-bearing NmAb sample and CFF alone were analyzed by MASC luminon assay (Figure 1B). Fluorescence detection was achieved using excitation and emission wavelengths of 450 and 520 nm, respectively. The (NmAb:P_as_^*^)_n_ aggregates elute at 3.2 min followed by the NmAb:P_as_^*^ monomer at 3.7 min (Figure 1B). The overlaid chromatograms of NmAb in CFF and CFF alone reveal that neither host-cell proteins nor metabolites hamper quantification of aggregates and monomers of NmAb after coding. The small peaks eluting between 5 and 8.5 min result from nonspecific binding of P_as_^*^ to host-cell proteins in CFF and do not interfere with NmAb quantification.

**Figure 2:**
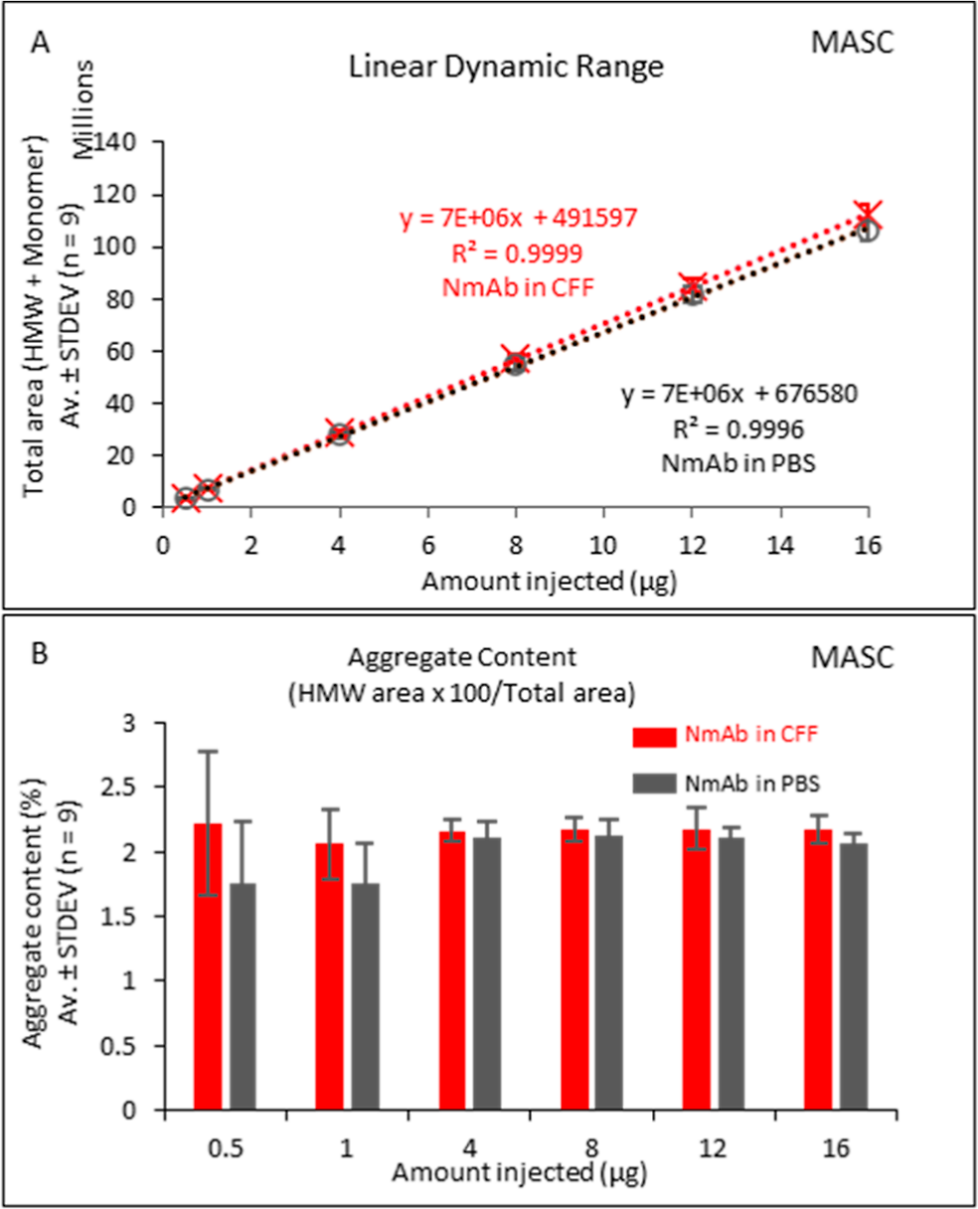
CFF components do not significantly affect the values for aggregate content and mAb titer in the MASC assay. NmAb samples were diluted to 1 mg/mL in PBS or CFF for analysis. (A) Linear dynamic range of mAb titer analysis by the standard MASC method. (B) Aggregate content for different amounts of injected NmAb by the standard MASC method.

It is significant that the traditional method for aggregate quantification by SEC using absorbance detection at 280 nm did not differentiate between host-cell proteins and NmAb in the 2–5 min elution time-window (inset, Figure 1A). In contrast, after fluorescent coding of the NmAb proteoforms, the host-cell proteome and metabolome were no longer detected in the MASC luminon assay (Figure 1B). Differential coding of mAb proteoforms with a highly selective fluorescently coded affinity selector P_as_^*^ clearly enhanced the differentiation between mAb species and host-cell proteins. Together, the results shown in Figure 1 support the hypothesis that the structure-specific fluorescence coding and differing linear velocities of analytes, nonanalytes, and reagents are enabling features in MASC assays. The results also indicate that the fluorescent affinity selector encodes one or more structural features common to both monomer and aggregate mAb proteoforms. mAb titer is the sum of monomer and high molecular weight species concentrations.^32^ Aggregate content is normally expressed as a percent of the total mAb content. The linear dynamic range and percent aggregate content in MASC assays are not significantly impacted by the presence or absence of CFF in samples across the tested 32-fold NmAb range (Figure 2A,B). For NmAb samples formulated in PBS, the average aggregate recovery shows a 0.5% increase as the quantity of NmAb injected increases from 0.5 to 4 μg (Figure 2B). However, this increase is not statistically significant. The origin of this phenomenon, if real, is unknown.

**Figure 3:**
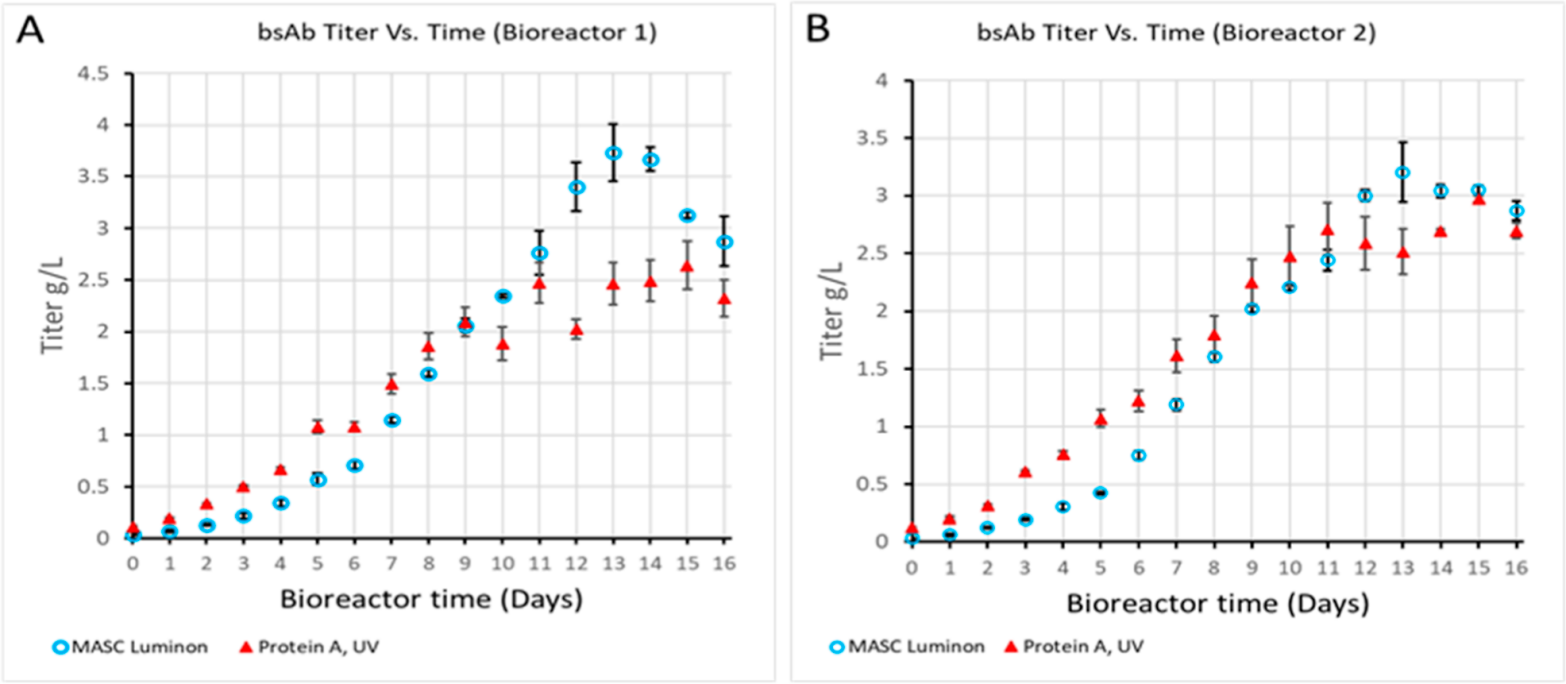
Comparison of the temporal profiles of titer in the CFF of a therapeutic bispecific antibody (bsAb) over a 16-day period in two single use 250-mL reaction vessels of an Ambr 250 multiparallel bioreactor system with the MASC luminon assay and UV absorbance following protein A purification. Each data point is the average ± STDEV of triplicate samples. (A) Bioreactor 1 and (B) bioreactor 2.

Comparison of the mAb titer in the CFF of a therapeutic bispecific antibody over a 16-day period in two bioreactor runs by the MASC luminon assay as well as by protein A (Figure 3) shows a good correlation between the MASC luminon assay and the traditional titer method. Besides the titer and relative aggregate content, the MASC luminon assay is able to monitor the content of monomer and various aggregate species (dimer, trimer, and tetramer) of a therapeutic bispecific antibody directly from CFF over consecutive bioreactor time points (Figure S1). Thus, notable features of the MASC luminon assay are its simplicity, ability to deliver titer and detailed aggregation profile within 5 min, analytical cycle times of less than 10 min, and elimination of the need for host-cell proteome and metabolome removal prior to analysis.

**Figure 4:**
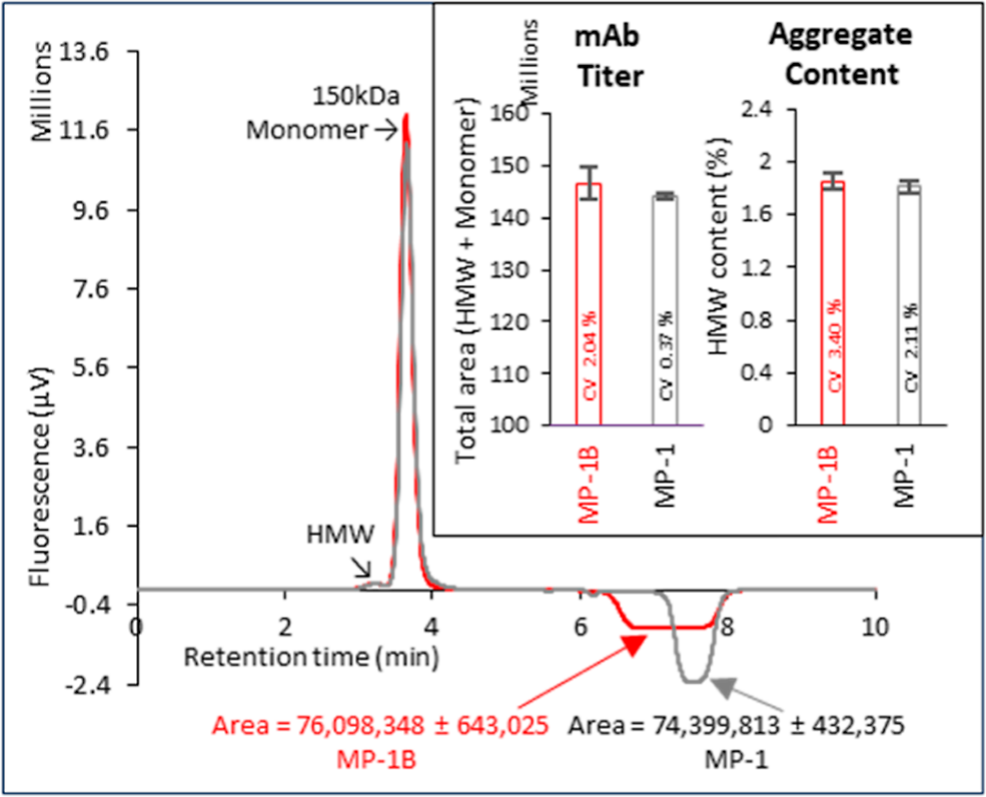
Effect of the affinity selector (P_as_^*^) concentration in the mobile phase on the MASC chromatogram. Shown is an overlay of MASC chromatograms of NmAb performed under standard conditions with the customary concentration of affinity selector in the mobile phase (MP-1, 100% P_as_^*^ conc.; *gray*) and half-strength affinity selector in the mobile phase (MP-1B, 50% P_as_^*^. conc.; *red*). The effect of mobile phase affinity selector concentration on mAb titer and aggregate content is shown (inset). Sample, NmAb in PBS (16 μg; *n* = 3).

To test the hypothesis proposed in the “Assay Design” section that the A_p_:P_as_^*^ complex is formed continuously during flow, peak widths in the MASC luminon assay were compared for a 16 μg injection of NmAb with the standard (100%) and half strength (50%) concentration of P_as_^*^ in the mobile phase (Figure 4). Reducing the concentration of the affinity selector in the mobile phase did not affect the total area of the mAb peaks or the percent aggregate content.

At the lower P_as_^*^ concentration, depletion of P_as_^*^ from the mobile phase, due to complexation with the analyte A_p_, continues to occur for a longer time as A_p_ moves through the column into fresh P_as_^*^. This is indicated by the greater trough width in the chromatogram at the lower P_as_^*^ concentration. Together, these results indicate that the A_p_:P_as_^*^ complex is formed continuously and the coding is complete. The surprise is in reproducibility. Both the titer and aggregate content are of poorer reproducibility at a lower reaction rate (Figure 4, inset). Diminished reproducibility could also arise from adsorption of proteins. Rapid coding is therefore a desirable condition for the MASC luminon assay.

### Assay Selectivity

**Figure 5:**
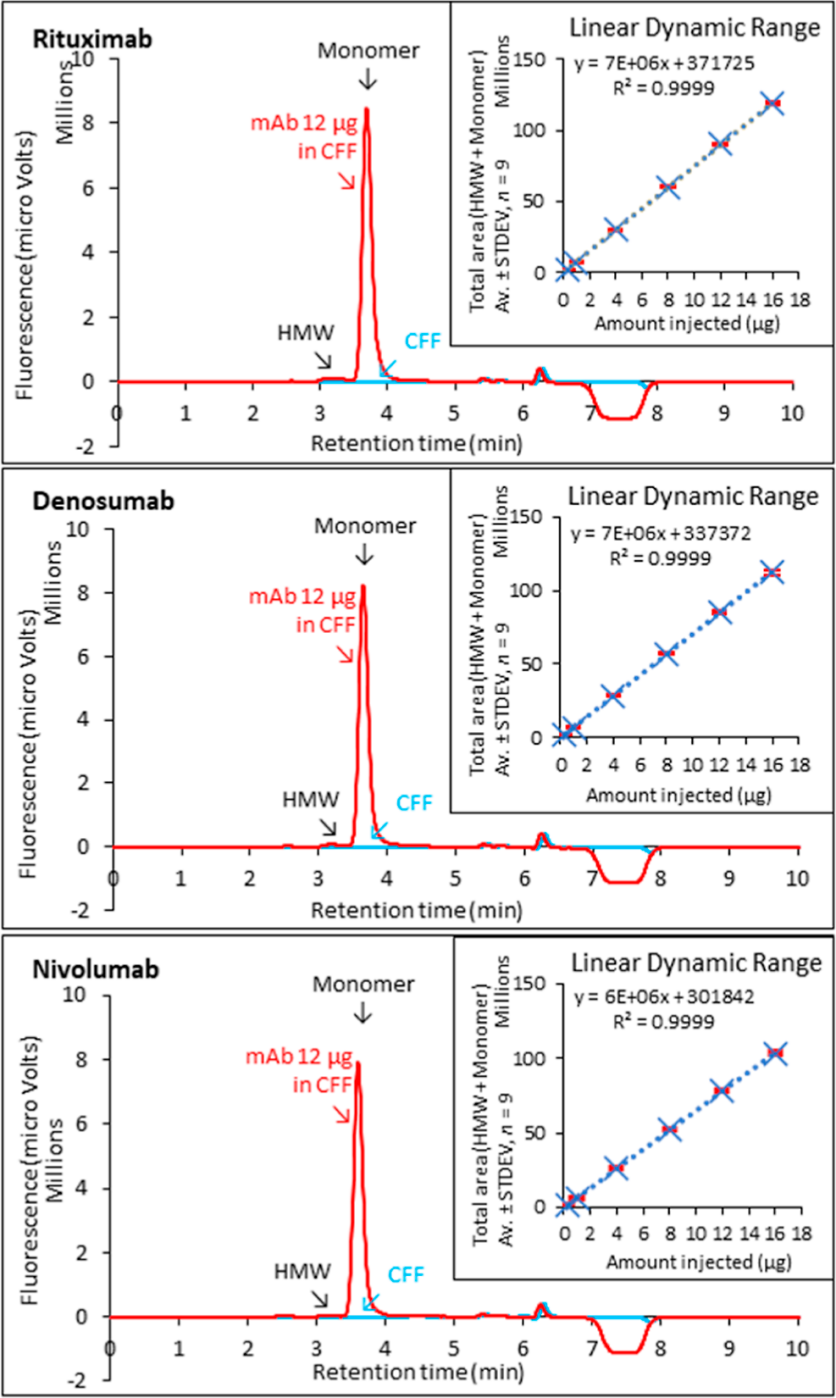
Titer analysis of biosimilars by MASC. Shown are the representative chromatograms and the linear dynamic range (inset) for biosimilars of rituximab (IgG1), denosumab (IgG2), and nivolumab (IgG4 S228P). Mobile phase L-MP from Proteometer-L kit. Flow rate, 1 mL/min. Detection method, fluorescence Ex. 450 nm and Em. 520 nm. mAb samples were diluted to 1 mg/mL in CFF for analysis (red lines). An equal volume of CFF alone was injected for comparison (blue lines). Data are from three independent experiments with triplicate samples.

Rituximab, denosumab, and nivolumab analyses show that the MASC luminon assay quantified the titer and percent aggregate content of these IgG1κ, IgG2, and IgG4 subclasses of fully human and chimeric mAbs in the presence of host-cell proteome and metabolome (Figure 5 and Table 1). The linear dynamic range for NmAb, rituximab, denosumab, and nivolumab is the same, that is, 0.5–16 μg (Figures 2 and 5). Differences in the total area for a constant amount of mAb injected are attributed to the structural dissimilarities in these antibodies that alter the P_as_^*^ fluorescence (Table 1). The aggregate content depends on the mass of mAb injected (Figure S2). Injections of mAb in amounts of approximately 12 μg gave the most consistent values (Table S1). The coefficient of variation (CV) for aggregate content is greater than the CV for titer, as expected since aggregates constitute a very small fraction of the mAb samples (Tables 1 and S1). When the retention times of the peaks and P_as_^*^ subtraction trough (Figures 1, 5, and Table 1) are considered collectively, it is further concluded that the selectivity, binding rate, and binding strength of P_as_^*^ were roughly the same for the four mAbs tested.

**Table 1:** Results of MASC Reproducibility Tests

MASC reproducibility sample = mAb in CFF (4 μg)								
					retention time (min)			
	total area (HMW + monomer)		aggregate content (%)		HMW		monomer	
mAb	average ± STDEV	CV (%)	average ± STDEV	CV (%)	average ± STDEV	CV (%)	average ± STDEV	CV (%)
NmAb (*n* = 30 injections)	29,180,175 ± 856,822	2.94	2.19 ± 0.098	4.06	3.22 ± 0.007	0.22	3.66 ± 0.000	0.00
Rituximab (*n* = 24 injections)	30,421,387 ± 308,664	1.01	2.15 ± 0.071	3.31	3.09 ± 0.006	0.18	3.70 ± 0.000	0.00
Denosumab (*n* = 24 injections)	28,767,664 ± 407,264	1.42	1.26 ± 0.074	5.87	3.17 ± 0.009	0.30	3.65 ± 0.003	0.07
Nivolumab (*n* = 24 injections)	26,326,987 ± 263,368	1.00	0.177 ± 0.039	22.3	3.12 ± 0.021	0.69	3.59 ± 0.002	0.06

These data also indicate that the binding site of P_as_^*^ is a structural feature shared by IgG1, IgG2, and IgG4. Accordingly, P_as_^*^ binds to the Fc region of human IgG, a structure shared by the three tested biosimilars and all therapeutic mAbs (Table S2). When considering the IgG binding characteristics of protein A, protein G, and protein L, it is evident that the specificity of P_as_^*^ has some similarities and differences. For example, protein A and protein G also bind to the Fc region of these three IgG subclasses. However, protein A binds weakly to human IgA, IgM, and Fab, but P_as_^*^ does not (Table S2). P_as_^*^ binding to nonhuman IgGs is either poor or insignificant in all cases tested except those of porcine and equine origin. Unlike the strong binding of protein A to canine and mouse IgGs, P_as_^*^ binding is insignificant. It appears that the selectivity of P_as_^*^ for human IgGs exceeds that of protein A and protein G. A different low molecular weight affinity selector would need to be developed for MASC luminon assays of nonhuman antibodies.

### Linear Dynamic Range and Reproducibility

The linear dynamic range for mAb quantification depends on the fluorescence detector and its settings. Routine sensitivity in our systems ranged from 0.5 to 16 μg of protein employing the factory default setting of Gain 4x and medium sensitivity (Figures 2 and 5). The range can be adjusted by altering the fluorescence detector settings. The slope of the standard curve in LDR assays exhibits a sample matrix effect, being slightly higher for NmAb in CFF relative to NmAb in PBS or l-His buffer (Figures 2 and S3). The slope of the standard curve is a characteristic of the mAb molecule (Figure S3). The data show that NmAb or preferably the analyte test mAb in a buffer can serve as a standard for monitoring titer changes or comparing mAb titer in different fermentors. Ideally, every mAb requires its own standard curve in the appropriate sample matrix to obtain the correct titers.

With 24–30 identical injections (4 μg) of CFF bearing NmAb, rituximab, denosumab, or nivolumab, the retention times of their aggregate and monomer peaks as well as total peak areas are very reproducible in three single day trials (Table 1). Coefficients of variance (CV) of less than 1% are observed for the retention times, and the CV of total peak area ranges from 1.00 to 2.94% (Table 1). Even for different injected amounts, the CV of total area for triplicate injections over three independent experiments is less than 2% for all samples (Table S1). Measured titer values with NmAb amounts in the lower, middle, and upper linear dynamic ranges lie between 90 and 94% of the expected value with CVs ranging from 0.01 to 0.27% over the tested amounts (Table S3). In comparison, a 6.9% CV has been reported for LC–MS analyses of a therapeutic mAb following extensive sample preparation and signature peptide-based quantification by multiple reaction monitoring.^33^

Aggregate content is known to vary depending on factors such as mAb concentration, and buffer composition.^34^ Accordingly, the aggregate content as measured by the MASC luminon assay is dependent on the amount of mAb injected (Figure S2). Accurate comparisons of aggregate content therefore require equivalent amounts of mAb. Reproducibility of aggregate content ranges from CVs of 3.31 to 5.87% when 24–30 identical injections (4 μg) of rituximab, denosumab, or NmAb formulated in CFF were performed over the course of three different days (Table 1). However, a CV of 22% is obtained for aggregate content for nivolumab formulated in CFF under the same test conditions, possibly due to the lower aggregate content or aggregation potential. The aggregate content for NmAb in CFF from identical injections (4 μg), obtained from three different lots of the Proteometer-L kits and three different LCs, returned values of 2.15% (±0.21) with a coefficient of variance of 9.56% (119 runs total). The MASC luminon assay is able to quantify relative aggregate contents as high as 35–60% in a bispecific antibody in CFF and in samples containing cross-linked aggregates of NmAb formulated in PBS or CFF (Figures S1 and S4). Aggregate content observed in the cross-linked NmAb samples as determined by the MASC luminon assay and the UV absorbance were similar; however, the MASC luminon assay reported values near 91–95% of the UV absorbance values (Figure S4). This discrepancy is likely due to the differences in the relative response of aggregates and monomer between detection methods (UV vs fluorescence) and/or due to steric hindrance of P_as_^*^ reagent binding in aggregates.

## Discussion and Conclusions

Rapid identification and quantification of significant mAb quality features, namely, mAb titer and percent aggregate content in minutes, were addressed here through the MASC luminon assay. This is a new type of molecular recognition assay that shares some similarities with online 2D-LC protein A-SEC assays. It is comparable to online 2D-LC protein A-SEC for speed and the ability to estimate mAb titer and relative aggregate content directly in CFF.^35^ However, the MASC luminon assay offers improved accuracy and reproducibility owing to elimination of the first dimension mAb purification step by protein A chromatography. Specifically, variability in mAb monomer and aggregate ratios arising from (i) duration of exposure to and composition of the acidic protein A elution buffer, (ii) band broadening in the first dimension, (iii) selection of the collection window and sample volume of protein A-eluted mAb, and (iv) mAb adsorption on the inner surface of the sample loop for the second dimension LC are eliminated.

The MASC luminon assay is a new form of process analytical technology that enables the preparation of cellular extracts for analysis, analyte-specific fluorescent coding, and proteoform separation based on size in a single isocratically eluted column. A major attribute of this assay format is that mAb quality assessment could be based on the resolution and detection of fluorescently coded mAb monomer and aggregates in fermentor samples without the universally used preliminary mAb purification by protein A affinity chromatography.^36^ Fluorescent coding of a constant region of human mAbs with high specificity circumvents the need for preliminary removal of the host-cell proteome and metabolome from samples while making the assay broadly applicable to all therapeutic mAb subclasses. Although the MASC luminon assay format provides no structural information beyond targeting the constant Fc region of mAbs for detection, it provides value in enabling the creation of a chronological pattern of monomer to aggregate ratios. When coupled to a fluorescence detector, mAb titer and percent aggregate content were quantified in 10 min or less, independent of their composition of other CQAs. Aggregate-to-monomer ratios are very important in assessing the risk of mAb toxicity and immunogenicity. The MASC luminon assay can thus provide rapid and timely early warning of mAb quality drift, which requires higher level validation and remediation.

It is concluded that the MASC luminon assay protocol described here is more suited to assess mAb function and higher order structure than critical structure attributes within the primary and secondary structures of mAbs. The MASC luminon assay can rapidly identify the incidence of protein quality problems for subsequent in-depth analysis by LC–MS methods. The simplicity and speed of the MASC luminon assay enables data-dependent decision making, which is the holy grail of therapeutic mAb quality management during process development and manufacturing. Moreover, this assay technology can be used with multiple IgG subclasses and allotypes of human or humanized antibodies. The potential for parallel coding and detection of multiple CQAs in a proteoform family suggests that MASC luminon assays will be a powerful new addition to process analytical technology.

## Supporting Information Available

Results of MASC robustness tests, specificity of the MASC affinity selector, results of MASC reproducibility and accuracy tests, bsAb titer and aggregate over a 16-day bioreactor run, aggregate analysis of biosimilars, mAb titer slope comparisons in different matrices, and aggregate content range analysis (PDF)
