# Supplementary material for: Mobile Affinity Selection Chromatography Analysis of Therapeutic Monoclonal Antibodies
Source: Anal Chem. 2023 Oct 26;95(44):16115–22. doi: 10.1021/acs.analchem.3c02180 (PMC10633814; doi:10.1021/acs.analchem.3c02180)
Supplement: Supplementary file 1 — ac3c02180_si_001.pdf [file ac3c02180_si_001.pdf]

Supporting information for

Mobile Affinity Selection Chromatography Analysis of Therapeutic Monoclonal Antibodies

Authors: Meena L. Narsimhan[1], Jinhee Kim[1], Nathan A. Morris[1], Mary A. Bower[1], Harsha P. Gunawardena[2], Eric Bowen[1], Fred E. Regnier[1*]

Author affiliations:

[1]Novilytic, LLC, 1281 Win Hentschel Boulevard, West Lafayette, IN 47906, USA

[2]Janssen Research & Development, The Janssen Pharmaceutical Companies of Johnson & Johnson, 1400 McKean Road, Spring House, PA 19477, USA

[*]Corresponding author: fregnier@novilytic.com

Table of contents

Table S1 ____ S2

Table S2 ____ S3

Table S3 ____ S4

Figure S1 ____ S5

Figure S2 ____ S6

Figure S3 ____ S7

Figure S4 ____ S8

Table S1. Results of MASC robustness tests

MASC LDR Assays Robustness									
mAb (*n* = 9) injections)	Total Area (HMW + Monomer) CV (%)					Aggregate Content CV (%)			
Amount (ug)	1	4	8	12	16	4	8	12	16
NmAb in PBS	2.00	1.30	1.15	0.99	0.99	5.33	2.53	2.01	10.4
NmAb in CFF	1.38	1.19	1.37	1.11	1.58	4.84	9.21	4.17	10.6
Rituximab in CFF	1.34	1.01	0.96	1.08	1.38	2.45	4.33	3.73	8.70
Denosumab in CFF	1.82	1.24	1.13	1.11	1.15	8.05	3.81	4.40	3.63
Nivolumab in CFF	0.64	0.69	0.74	0.81	0.88	23.8	19.0	14.2	12.4

Table S2. Specificity of the MASC affinity selector

Species	Subclass	Luminon*	Protein A**	Protein G**	Protein L**
Human	Total IgG	S	S	S	S
	IgG1	S	S	S	S
	IgG2	S	S	S	S
	IgG4	S	S	S	S
	IgM	ns	W	-	S
	IgA	ns	W	-	S
	Fab	ns	W	W	S
	Fc	S	S	S	-
Porcine	Total IgG	S	S	W	S
Equine	Total IgG	M	W	S	?
Goat	Total IgG	W	W	S	-
Bovine	Total IgG	W	W	S	-
Canine	Total IgG	ns	S	W	?
Mouse	Total IgG	ns	S	S	S

* Signal strength S = strong; M = medium; W = weak; ns = not significant

** Binding strength S = strong; M = medium; W = weak; - = not bound; ? = unknown

Table S3. Results of MASC reproducibility and accuracy tests

**MASC Accuracy Tests (*n* = 3 injections)<br>Test NmAb amounts correspond to lower, middle and upper values of linear dynamic range**			
Amount Expected (µg)	Amount Reported (µg)	Accuracy (%)	CV (%)
1.49	1.34	90	0.27
7.01	6.49	93	0.01
15.0	14.0	94	0.01

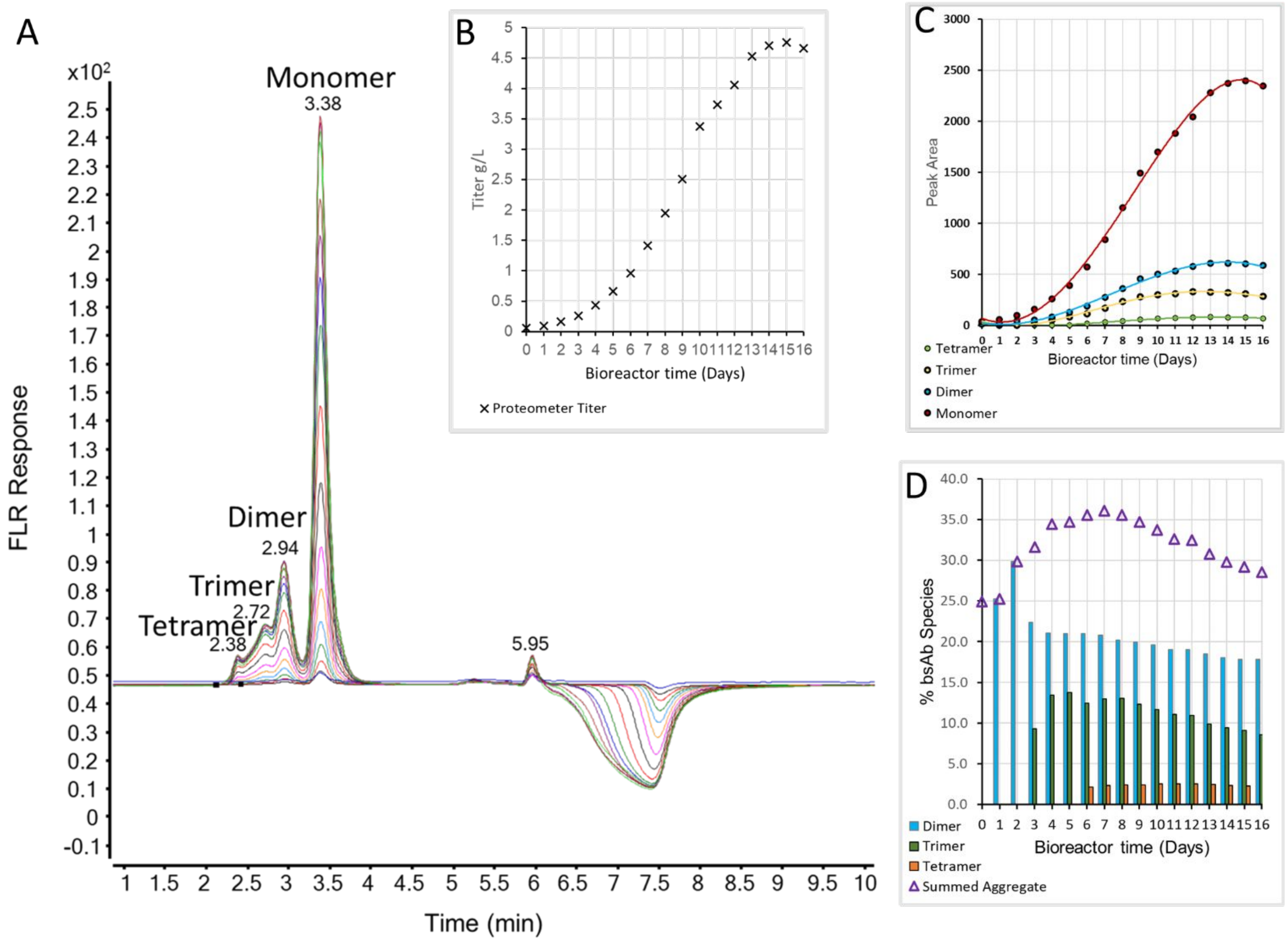

Figure S1. MASC monitoring of a therapeutic bispecific antibody (bsAb) titer and aggregation using Proteometer-L assay over the course of a single Ambr 250 bioreactor run. (A) FLR response Vs. retention time of 16 cell-free culture filtrates obtained over 16 consecutive bioreactor time points. Inset shows the peak areas of the monomeric, dimeric, trimeric, and tetrameric species of the bsAb derived from a single clone (B) bsAb titer at each bioreactor time point is estimatred using the slope of a regression analysis performed using NmAb calibration standard spiked into culture media (C) Time course of peak areas of the monomeric, dimeric, trimeric, and tetrameric proteoforms (D) Summed aggregation and aggregate composition profile of the bsAb over the bioreactor time course estimated using peak areas.

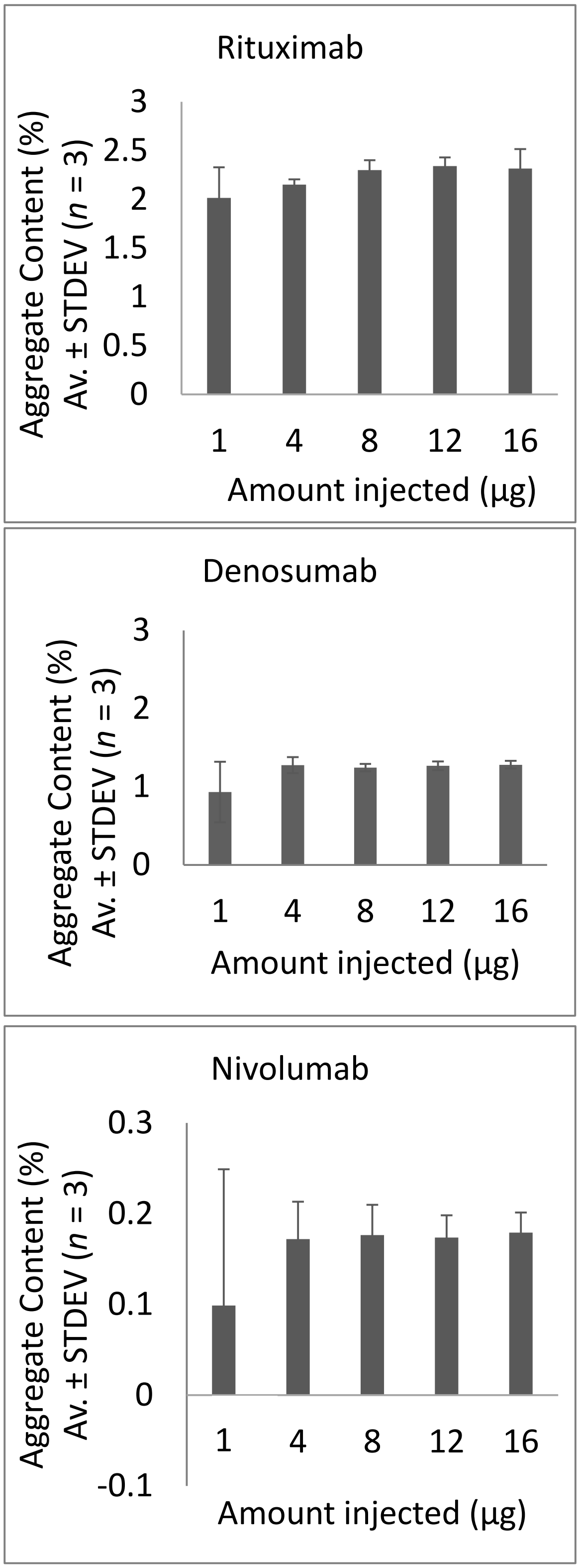

Figure S2. Aggregate analysis of biosimilars by MASC. Shown are results of aggregate content as a function of the amount of mAb injected over the linear dynamic range of biosimilars of Rituximab (IgG1κ), Denosumab (IgG2) and Nivolumab (IgG4 S228P). Mobile phase , L-MP from Proteometer-L

kit. Flow rate, 1 mL/min. Detection method, fluorescence Ex. 450 nm Em, 520 nm. mAb samples were diluted to 1 mg/mL in CFF for analysis

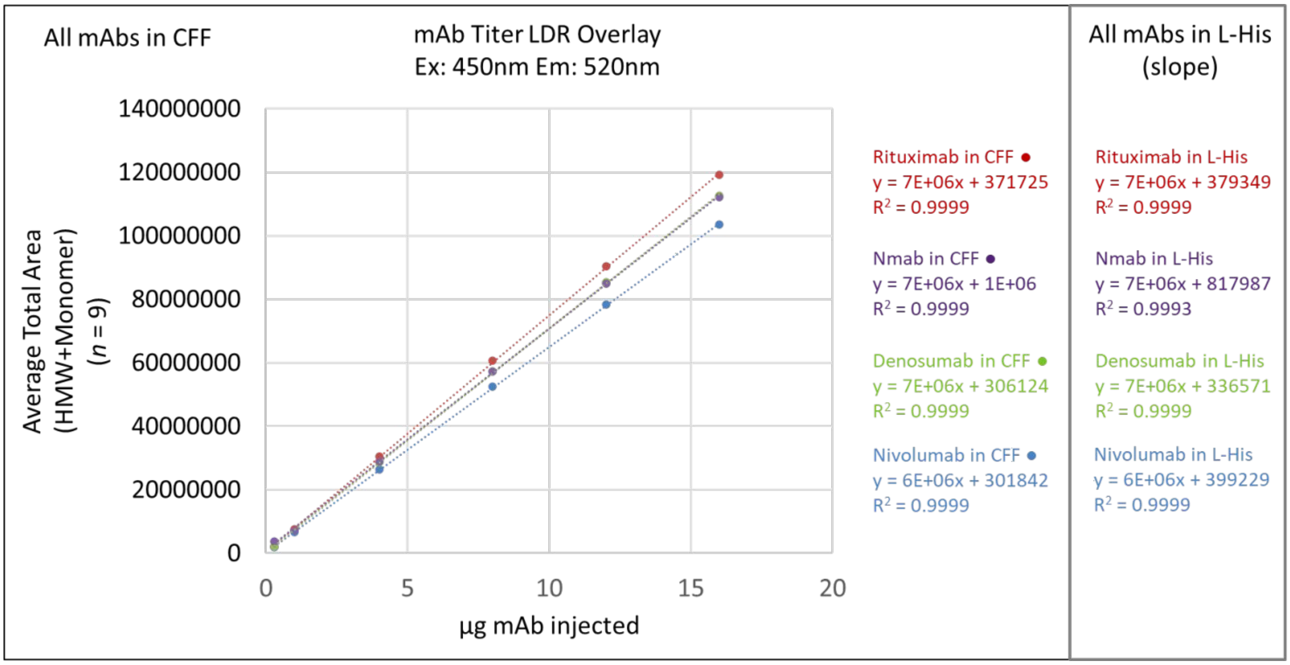

Figure S3. mAb solution in L-His buffer can be used as a standard for its quantification by MASC. Shown (left panel) are results of LDR analysis of biosimilars of Rituximab, Denosumab, Nivolumab and NmAb in CFF. The graph shows that every mAb has a distinct slope. However, the slope of each mAb in CFF was very close to the slope obtained in 12.5 mM L-His HCl buffer pH 6 as shown (right panel, graph not shown). All samples were injected from 1 mg/mL solutions.

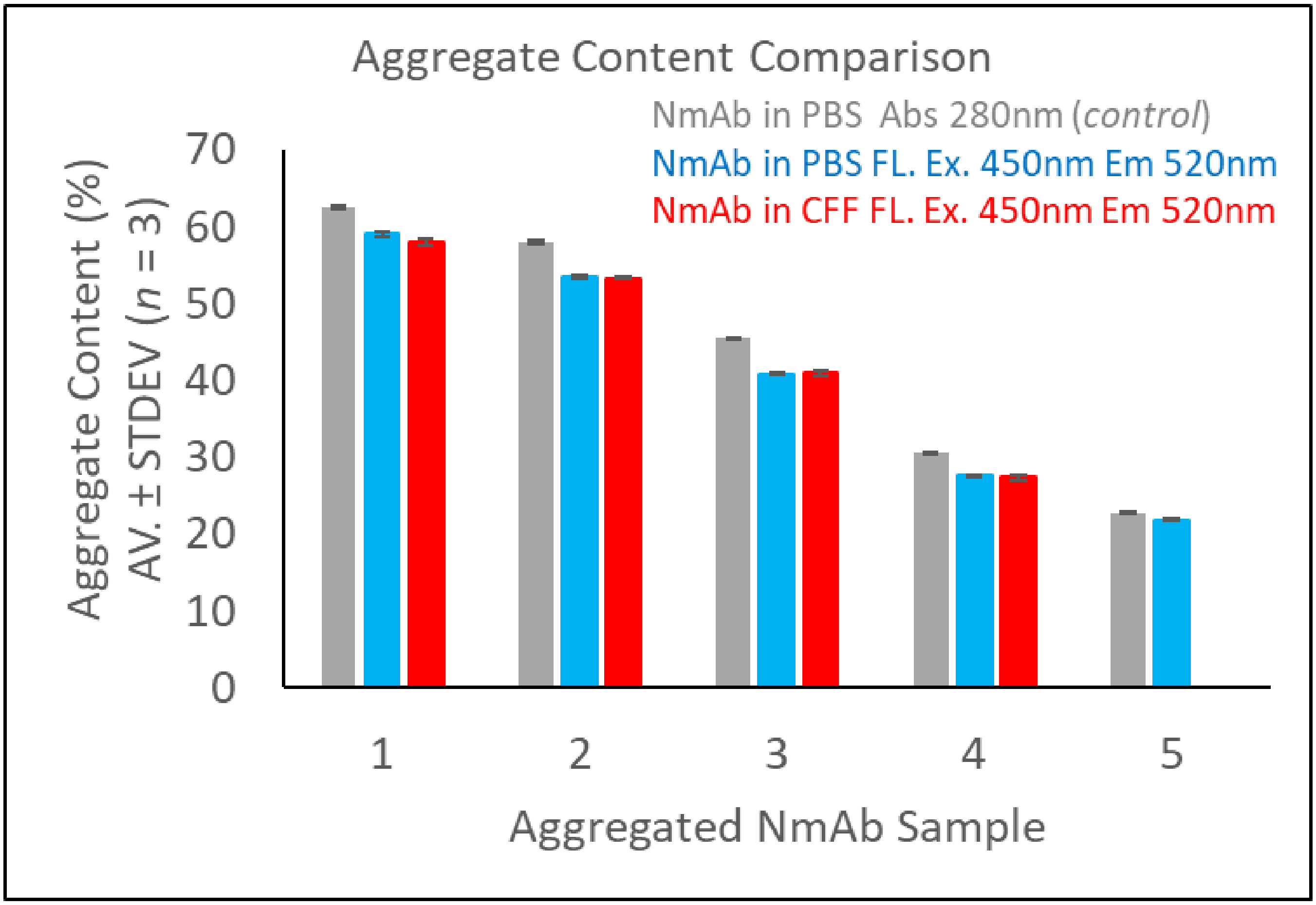

Figure S4. Applicability of the MASC method to high aggregate content samples. Shown are the results of aggregate content analysis by the standard MASC protocol from 4 µL (4 µg) aliquots of the aggregated NmAb dissolved in PBS or CFF. Also shown is a control, where aggregate content was measured by UV absorbance in the NmAb in PBS sample. The aggregate content of NmAb by MASC lay between 90 % and 94 % of the corresponding values obtained by UV absorbance. Samples 1 – 4 were NmAb at various concentrations in PBS that were crosslinked using BS3. Sample 5 was obtained by shear stress followed by crosslinking with BS3.

Method for Figure S4. Samples 1 – 4 were prepared by crosslinking NmAb in PBS at various starting concentrations (1, 2.5, 5, & 7.5 mg/mL). The crosslinking reaction was performed by addition of a 25 mM solution of BS3 [bis(succinimidyl)suberate] (ThermoFisher Scientific) in water to a final concentration of 5.2 mM. The reaction was allowed to proceed at room temperature for 30 minutes and then quenched with Tris HCl pH 7.5 to a final concentration of 21 mM. Samples were then buffer exchanged into PBS by ultrafiltration (Amicon Ultra-0.5, 100kDa MWCO) and made up to a final concentration of 1 mg/mL. These constitute the ‘NmAb in PBS’ samples. ‘NmAb in CFF’ samples were

created by adding ‘NmAb in PBS’ aliquots to a freeze-dried 100 µL aliquot of CFF. Sample 5 was obtained by subjecting a 1 mg/mL solution of NmAb in PBS to shear stress by vortexing (Vortex Genie 2T, speed 5) for 6 hours at room temperature, and then crosslinking as described above.
